# Assurance and Development of Interaction Quality: The Impact of Blended-Learning Professional Development Training Programme

**DOI:** 10.1007/s10643-023-01479-7

**Published:** 2023-04-15

**Authors:** Eva Pölzl-Stefanec, Mailina Barta, Catherine Walter-Laager

**Affiliations:** 1 Institute of Education Research and Teacher Education: Early Childhood Education, Research Unit, University of Graz, Graz, Austria; 2 International Centre for Professionalisation in Early Childhood Educational Practice, University of Graz, Graz, Austria; 3 International Centre for Professionalisation in Early Childhood and Practice, University of Graz, Graz, Austria

**Keywords:** Interaction quality, Early childhood education, Assessment, Online development training, Blended-learning

## Abstract

In recent years, there has been considerable investment at the European Union level in expanding early childhood education and care (ECEC) facilities. In line with this quantitative substantial initiative, research and social policies are increasingly focusing on the quality of such facilities. High quality depends, among other things, on well-trained early childhood educators. This poses a dilemma for early childhood educators for various reasons; there is a shortage of skilled early childhood professionals, so that low-skilled staff are also being employed in early childhood education facilities. Online formats for professional development can contribute to the professionalisation of the ECEC system through vocational training. Since these formats are designed and produced to high professional and technical standards, they can be cost-effective thanks to their multiple uses and because they can often be completed by participants independent of time and location. This article presents an empirically studied blended e-learning training format based on the principles of co-constructivist didactics. The content focuses on the quality of interaction between early childhood professionals and children. Before and after the training course was completed, standardised non-participant observations were conducted in Austrian, German, Hungarian, Slovenian, Italian, and Portuguese early childhood education and care institutions. The before/after measurements (N = 43) showed a significant effect on the quality of interaction between the early childhood professionals and the children.

## Introduction

National policies in European countries increasingly recognise the need to invest in early childhood education and care (ECEC) systems. This is no longer just about enabling parents to work, but also about contributing to equal opportunities in the socio-educational context (OECD, 2020a; Stamm & Edelmann, 2013). In the wake of this major initiative to expand crèches and kindergartens (European Union, 2019), the question of how to ensure and promote the quality of education is increasingly coming to the fore (Edwards, 2021).

The connection between quantitative expansion, high quality, and the availability of active early childhood professionals poses a dilemma for early childhood education. Especially in German-speaking countries, there is a serious lack of skilled workers (OECD, 2020a, 2020b). To alleviate the shortage, insufficiently qualified staff are often employed in early childhood education and care institutions (125. Änderung der Kinderbetreuungs-Ausbildungsverordnung [125th Amendment to the Child Care Training Ordinance 2010, 2020]; Baierl & Kaindl, 2021). This makes it increasingly difficult to ensure and improve the quality of early childhood education and care institutions.

### Professional Development Courses in the Context of Vocational Training

Studies have shown that besides ensuring low educator-to-child ratios and small groups of children, a high level of professional training, including in-service training, also contributes to the quality of early childhood education and care institutions (Burchinal, 2017; Early et al., 2007; Geißler et al., 2022). In continuing professional development (CPD) programmes, up-to-date knowledge is imparted, competencies for action are developed and reflection processes are conducted. The training of early childhood professionals is part of the formal education system in Europe and ranges from school education to Bachelor and Master degrees. CPD training is mostly voluntary (Buschle & Friedrich, 2020; Oberhuemer et al., 2010).

According to the OECD (2009) definition, CPD training involves professional activities that support individuals in improving their occupational requirements. The goal of CPD training is to further improve the quality of teaching in professional contexts (El Islami et al., 2022). CPD programmes are particularly effective when theory is successfully transferred to practice. Such programmes include at least 45–60 CPD hours and consist of regular seminars in eight-week units at a minimum (Egert & Kappauf, 2019).

Since attending CPD courses has been empirically shown to have aggregate effects on the knowledge, attitudes and behaviour of early childhood professionals (Fukkink & Lont, 2007; Werner et al., 2016), specific CPD courses could be a solution to the aforementioned dilemma. Moreover, studies focusing on pedagogical quality have found a correlation between the level of training and the quality of interaction between professionals and children (Geißler et al., 2022; Pianta et al., 2020; Sylva, 2010). Consequently, CPD programmes for early childhood professionals are seen as a glimmer of hope for professionalisation and quality development in early childhood education (Buschle & König, 2018). To counteract the rapid changes in working conditions, there is a need for innovative training and CPD programmes designed with the future in mind (Mertens, 1989).

### Online CPD in the Context of Early Childhood Education

Online professional development (OPD) formats can embrace innovative, future-oriented CPD concepts in the field of early childhood education. In general, the literature distinguishes between three types of e-learning formats: virtual learning platforms, which can be attended asynchronously; virtual classrooms, in which the content is delivered synchronously but online; and hybrid education spaces, which can be attended both on-site and online (Arnold et al., 2002; Salmon et al., 2015). Hybrid formats are often used synonymously with blended learning formats, as there is no clear definition for the latter. Nevertheless, blended learning is generally understood as a combination of face-to-face learning and asynchronous e-learning (Kim, 2007; Smith & Hill, 2019).

Online professional development programmes are not new to continuing vocational education; they have existed, mainly in English-speaking countries, since the 1980s (Archambault et al., 2022; Zawacki-Richter & Naidu, 2016). They have also been part of scientific research for more than 40 years. In Central Europe, OPD programmes are a novelty in the early childhood education context. Before the Covid-19 pandemic, there were hardly any OPD programmes for early childhood education in German-speaking countries. Not all early childhood education and care establishments provide ECE professionals with digital devices (e.g., PCs or tablets) and internet access. Moreover, digital skills are hardly part of early childhood educators’ training (Pölzl-Stefanec, 2021).

International studies have shown that online teaching and learning formats can be as effective as face-to-face learning (Askov et al., 2003; Doo et al., 2020; Drummond, 2011; Naidu, 2020; Salmon et al., 2015). Participants with high intrinsic motivation who can set goals, manage their time, and create learning plans to keep track of their progress are considered self-regulating learners who benefit most from ODP (Doo et al., 2020). Bonk (2011) surveyed participants in a massive online course for CPD and found that they mainly participated for the following purposes:

- Improving their professional knowledge in the context of a specific subject area
- Professional growth
- Self-improvement
- Satisfying their curiosity and general need for information

They also found the course beneficial for improving their professional skills, building confidence as learners, and helping others who needed similar knowledge. Since OPD programmes can be attended regardless of location and asynchronous programmes can be attended regardless of time, people from across a country or countries can participate without having to spend time and money travelling. Many learners can also attend standardized OPD programmes continuously (Reeves & Pedulla, 2011; Stone-MacDonald & Douglass, 2015). OPD programmes can help to introduce early childhood professionals in crèches and kindergartens to future-oriented vocational training. Innovative concepts based on didactic theories can contribute to quality assurance in early childhood education in a broader context (Pölzl-Stefanec & Geißler, 2022).

### Principles of Co-Constructivist Didactics in the Context of OPD Programmes

The fundamental competencies of OPD programmes in the twenty-first century are based on the principles of co-constructivist didactics, including learner-centredness and situated learning, building relationships and communities, integrating active learning, delegating responsibility to the participants, and personalising the learning process (Archambault et al., 2022). Co-constructivist didactics consists of interaction-oriented teaching and experiential and action-oriented methodological concepts. The ideas that all knowledge is constructed, that learning is an act of co-construction in a learning community and that teachers alone cannot produce learning are fundamental to co-constructivist thinking in didactics (Terhart, 2008). This means that people acquire knowledge and meaning from their experiences; they learn by combining new information with what they have already learned (Bada, 2015). Learning is understood as an active part. The active part is understood as a discovery process in which the learners are the main actors (Archambault et al., 2022). Trainers in OPD programmes do not create learning. They assume that participants have different interests, challenges and abilities, and they allow them to choose the order, topics, and tasks of the training activities (McCombs, 2015). This is especially true for OPDs, provided that the curriculum allows sufficient freedom.

The main guiding principles of co-constructivism are consideration of prior knowledge and experience, the process of actively building knowledge, connections and interactions between people and content, and time to experience and rethink concepts (Archambault et al., 2022). Programmes based on learning co-construction processes have a slightly different focus. Here, dialogue phases are deliberately built in to enable exchange within a learning community. Empirical studies have consistently shown that this approach is particularly effective in achieving specific learning goals (Lai, 2015; Nelson et al., 2008; Schrader, 2015). In this paper, we present an empirically studied OPD programme that can be considered a blended learning format and is based on the principles of co-constructivist didactics.

### Content, Aims, and Research Question of this Study

Recent studies (Paschall et al., 2022; Pianta et al., 2020) have focused on the quality of the interactions between early childhood education professionals and children in international contexts. This focus is based on fundamental theories in developmental psychology (Bowlby, 1982; Bronfenbrenner, 1997; Vygotsky, 1978). In the context of these theories, which are related to early childhood professionals, process quality, especially the availability and responsiveness of early childhood professionals, is a core aspect (Perlman et al., 2016). Early childhood educator-child interactions are the most common indicators of high process quality, which is known to promote children’s social, emotional, and cognitive development (OECD, 2018; Slot et al., 2018). Paschall et al. (2022) and Soliday Hong et al. (2019) summarized several studies showing that high-quality early childhood educator-child interactions are instrumental in promoting children’s language development, problem-solving skills, reading skills, and, subsequently, equity.

The Erasmus + Cooperation Partnerships project 'Qualimentary—Implementation of quality development processes in early education and care institutions' focused on the performance of early childhood professionals in their interactions with children. In this context, the term 'performance' refers to 'actions actually performed' (Fröhlich-Gildhoff et al., 2014) by early childhood professionals. Early childhood professionals from six European countries (Austria, Germany, Hungary, Slovenia, Italy, and Portugal) participated in an OPD programme in a blended learning format (2020–2021). The content of the programme was based on criteria for interaction quality defined in a previous project (Pölzl-Stefanec et al., 2020; Walter-Laager et al., 2018). The performance of the early childhood educators participating in the OPD programme was assessed before and after attending the OPD programme on site in the early childhood educational institutions by means of non-participatory observation using a standardised observation instrument (GrazIAS: Grazer Interaktionsskala für Kinder in den ersten sechs Lebensjahren) [Graz interaction scale for children in the first six years of life]. The observation instrument GrazIAS contains 12 characteristics for assessing the quality of interaction and is described in more detail in the following subchapter. This study addresses the following research question: *How does participation in an OPD programme in a blended learning format change the performance of professionals in their interactions with children in early childhood education and care institution settings?* The aim of the study is to ensure and further develop the quality of interaction between early childhood educators and children in nurseries and kindergartens after completing the OPD programme.

### Survey Methods: Quality Measurement

**Fig. 1:**
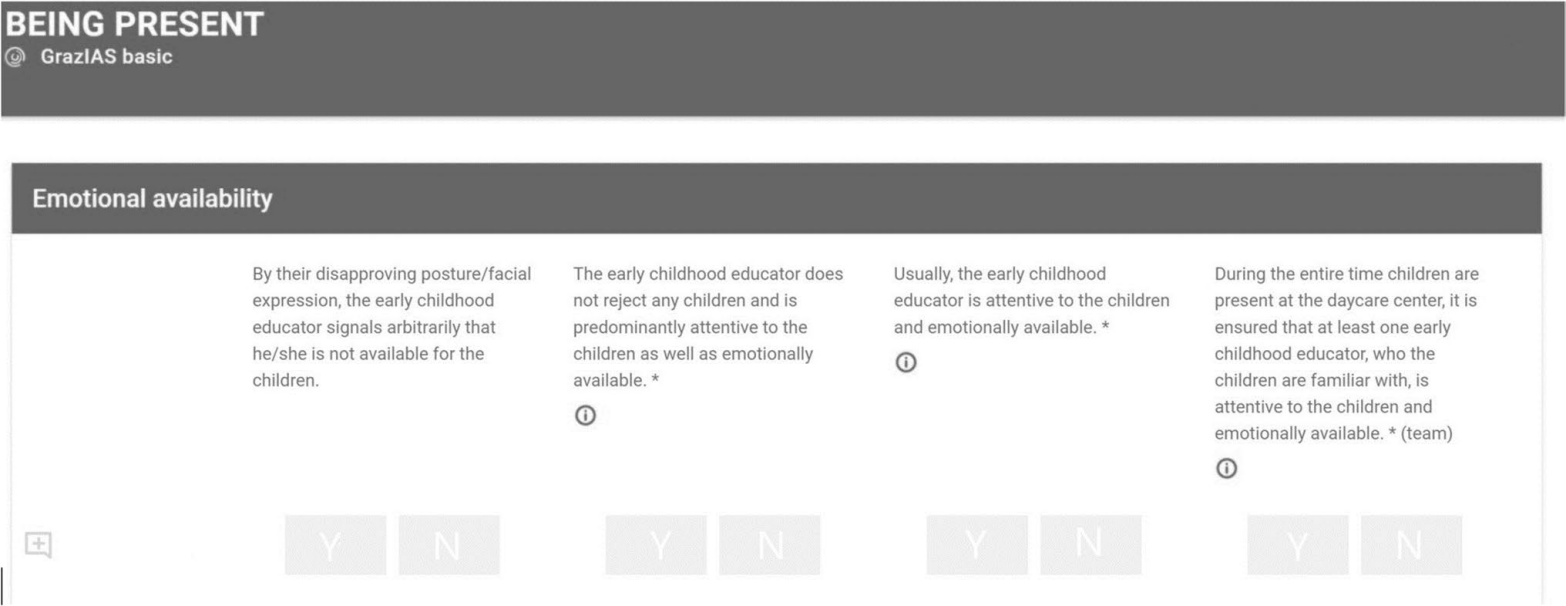
Example from the GrazIAS (Walter-Laager et al., 2018) criterion of being present, indicator: emotional availability, every indicator has four markers

A standardised observation instrument (GrazIAS) was used to measure the performance of each participating early childhood professional in interacting with children before and after completion of the OPD programme. The instrument was developed in recent years on the basis of current international scientifically based findings and validated by experts from science and practice (Walter-Laager et al., 2018). Indicator descriptions are used to measure characteristics of age-appropriate, developmentally supportive interaction quality. Figure 1 shows an example of the structure of the scale. The indicator descriptions focus on the design of good teaching–learning processes, especially the relationship design, and the children’s well-being (Remsberger, 2013; von Suchodoletz et al., 2014; Wadepohl & Mackowiak, 2016). These aspects are rated by trained assessors in four to five-hour non-participatory observations as *insufficient quality*, *minimal quality*, *good quality*, and *excellent quality*. The ratings are supplemented by qualitative descriptions (Walter-Laager et al., 2019).

The criteria for interaction quality in the GrazIAS observation instrument are divided into two subscales: 'Ensure relationships and well-being' (17 aspects for versions 0–3 years; 20 aspects for versions 3–6 years) and 'Support learning' (19 aspects for versions 0–3 years; 21 aspects for versions 3–6 years). Flöter et al. (2023a) examines the psychometric criteria of the subscales using three different samples and obtains Cronbach’s alpha values between 0.850 and 0.929 for the subscale 'Ensure relationships and well-being' and between 0.849 and 0.869 for the subscale 'Support learning'. Table 1 gives an overview of the distribution of the characteristics in the subscales 'Ensure relationships and wellbeing' and 'Support learning'.

**Table 1:** Overview of the criteria for interaction quality divided into the subscales 'Ensure relationships and well-being' and 'Support learning'

Ensure relationships and well-being		Support learning	
0–6 years	Being present	0–6 years	Enabling participation
0–6 years	Experiencing relationships	0–3 years	Offering and allowing sensory experiences
0–6 years	Introducing rules and adhering to them	0–6 years	Providing stimuli (verbally/non-verbally)
0–6 years	Supervising conflicts	0–3 years	Communicating in a stimulating way
0–6 years	Considering individual needs	3–6 years	Designing long-lasting dialogues
0–6 years	Supporting the regulation of emotions	3–6 years	Expanding vocabulary based on experiences
		3–6 years	Using language-promoting questions
		3–6 years	Shaping language

One to two individuals per project country received a 4 day online training in using the measurement instrument. After completing the training, a reliability test was performed using video examples from early childhood education practice. The participants had to achieve at least 85% inter-rater reliability agreement before they were allowed to perform quality measurements on site. The innovative GrazIAS digital application was used in the surveys to reduce errors, save resources and provide a uniform dataset.

### Participant Sample

Before the OPD programme started, private and public providers of early childhood education and care institutions in Austria, Germany, Hungary, Slovenia, Italy, and Portugal were invited to participate in a project on the topic of 'Ensuring and Improving the Quality of Interaction'. A total of 517 early childhood professionals working in early childhood education and care institutions were contacted in six countries. A prerequisite to participate in the OPD programme was that participants had a laptop or mobile device with an internet connection, camera, and microphone at their disposal. Participation was voluntary, and the participants had the right to withdraw at any time. This means that all participants who wanted to take part in the OPD programme could participate—no selection was made beforehand, the number of participants was not limited and the participants were not given any additional incentive to take part in the OPD programme. They just received a certificate of completion upon finishing the training. Following an explicit invitation to participate, a total of 84 early childhood professionals accepted the invitation to participate in the OPD program (Austria = 15, Germany = 9, Hungary = 18, Slovenia = 8, Italy = 10, Portugal = 24). 43 people (Austria = 9, Germany = 7, Hungary = 4, Slovenia = 8, Italy = 5, Portugal = 10) completed the course and participated in a survey both before and after the programme. This dropout rate (51%) may seem quite high, but it is usual for online professional development courses and is relatively low compared to other studies (up to 75%) (Gassler et al., 2004; Kamilali & Sofianopoulou, 2015). The dropout rate was related partly to the outbreak of the COVID-19 pandemic in 2020 and the associated challenges for educational professionals (Flöter et al., 2023b), and partly to the fact that the interval between the first and second standardised non-participant observations (February 2020 and May 2021) was more than a year. During this period, 13 participants withdrew from the OPD due to lack of time and 15 of the participating educational professionals went on parental leave or changed careers. 15 participants only took part in the asynchronous part of the training and could therefore not be included in the calculations. The sample includes all persons who participated in both the before/after measurements on site as well as in the asynchronous and synchronous parts of the OPD programme. At the beginning of the OPD programme, 18 of the 43 participating EC professionals worked in the nursery and 25 in the kindergarten (see Table 2). During the completion of the OPD programme, three participants changed from nursery to kindergarten. Three participants had less than three years of work experience. While 15 people had been working in ECE institutions for three to ten years, the majority of the participants (N = 19) had more than 10 years of professional experience, and 12 participants even have 20 years of professional experience. The basic education of the professionals ranges from upper secondary education to a Bachelor's degree and a Master's degree in early childhood education.

**Table 2:** Overview of participants before and after completion of the OPD programme measure

	Before^1^	Nursery	Kindergarten	After^1^	Nursery	Kindergarten
Austria	9	3	6	9	2	7
Germany	7	7	0	7	6	1
Hungary	4	0	4	4	0	4
Slovenia	8	2	6	8	4	4
Italy	5	2	3	5	2	3

### Description of the Blended Learning Setting

**Fig. 2:**
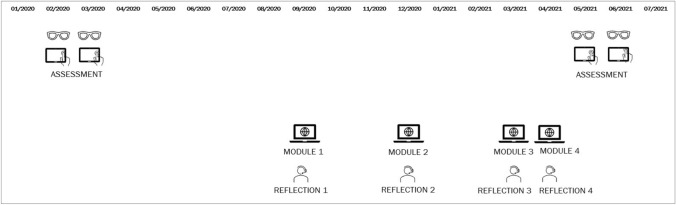
Schedule and elements of the online professional development programme. Modules 1, 2, 3, and 4: asynchronous part of the training; reflections 1, 2, 3, and 4 = synchronous part of the training

The vocational training took place between autumn 2020 and spring 2021 and consisted of 20 theoretical modules (50% of the training), which the participants completed asynchronously online, and four joint reflection sessions (30% of the training), which the participants attended synchronously using a *video conferencing* service. Figure 2 shows the timeline and elements of the blended learning OPD programme. All training content was available in English, German, Hungarian, Slovenian, Italian, and Portuguese. The performance of each early childhood professional’s interactions with children was measured before and after the training programme. After the programme, the participants received individual coaching, including feedback (20% of the training). The individual coaching is not part of the study design and took place after the second non-participant observation, it is not described in more detail in this article. There was no difference between the two surveys—they were conducted with the same instrument by the same project staff. The OPD programme involved a workload of 32 h for the participants. The 43 participants attended the blended learning training in the period from autumn 2020 to spring 2021.

The online training measure was designed using a blended learning format. All parts (theoretical inputs and reflection rounds) were attended online by the participants. The theoretical modules were completed in an asynchronous setting on a learning platform (Moodle), while the reflection sessions were attended in a synchronous setting via a conferencing tool. Table 3 shows the structure of the theoretical modules. Each of the 20 modules followed the same structure. The theory part, which is closely related to the topic of interaction quality, consists of a total of 20 modules. All training content was translated into English, German, Hungarian, Slovenian, Italian and Portuguese and the ECEs could participate in the OPD programme in their own language.

**Table 3:** Structure and methods of the theoretical modules

Structure		Principles of co-constructivist didactics	Methods
1	Introduction to the topic	Incorporation of prior knowledge and experience	Reflection questions, mind map
2	Theoretical input	Process of actively building knowledge	Technical text on the module topic
3	Interaction	Process of actively building knowledge	Quiz, forum discussion with other participants
4	Good practice video	Process of actively building knowledge	Watching a 3–8 min video
5	Reflection exercise	Connection to and interactions with other participants	Quiz, forum discussion with other participants
6	Transfer task	Time and space to reflect on what has been learned	Learning diary

Two theoretical modules were assigned to each participant based on the first on-site quality measurement. These covered areas where the participants needed further development. Each participant also chose two modules according to their individual training needs, interests, challenges, and skills. They were able to choose the order and timing of completing the modules. One trainer per country assisted the participants with technical or content-related issues, supervised the Moodle course discussion forums and accompanied the group during the reflection sessions. The reflection rounds, in which all 20 modules were discussed and worked on with the participants, took place synchronously. The 20 theoretical modules were designed according to the principles of co-constructivist didactics and were all structured in the same way.

The four joint reflection sessions took place at fixed times. During these sessions, the participants discussed the theory of the 20 modules and the developed contents with the trainer. The reflections were based on the notes that the participants had made in their learning diaries.

## Results

Because the data did not show a normal distribution, non-parametric methods were used in the analysis (Bortz et al., 2008). All statistical analyses were carried out with the software IBM SPSS Statistics version 27. Since the sample (N = 43) consisted of measurements taken in Austria, Germany, Hungary, Slovenia, Italy and Portugal, each project country formed a small sub-sample. Therefore, no country-specific analyses were carried out.

**Fig. 3:**
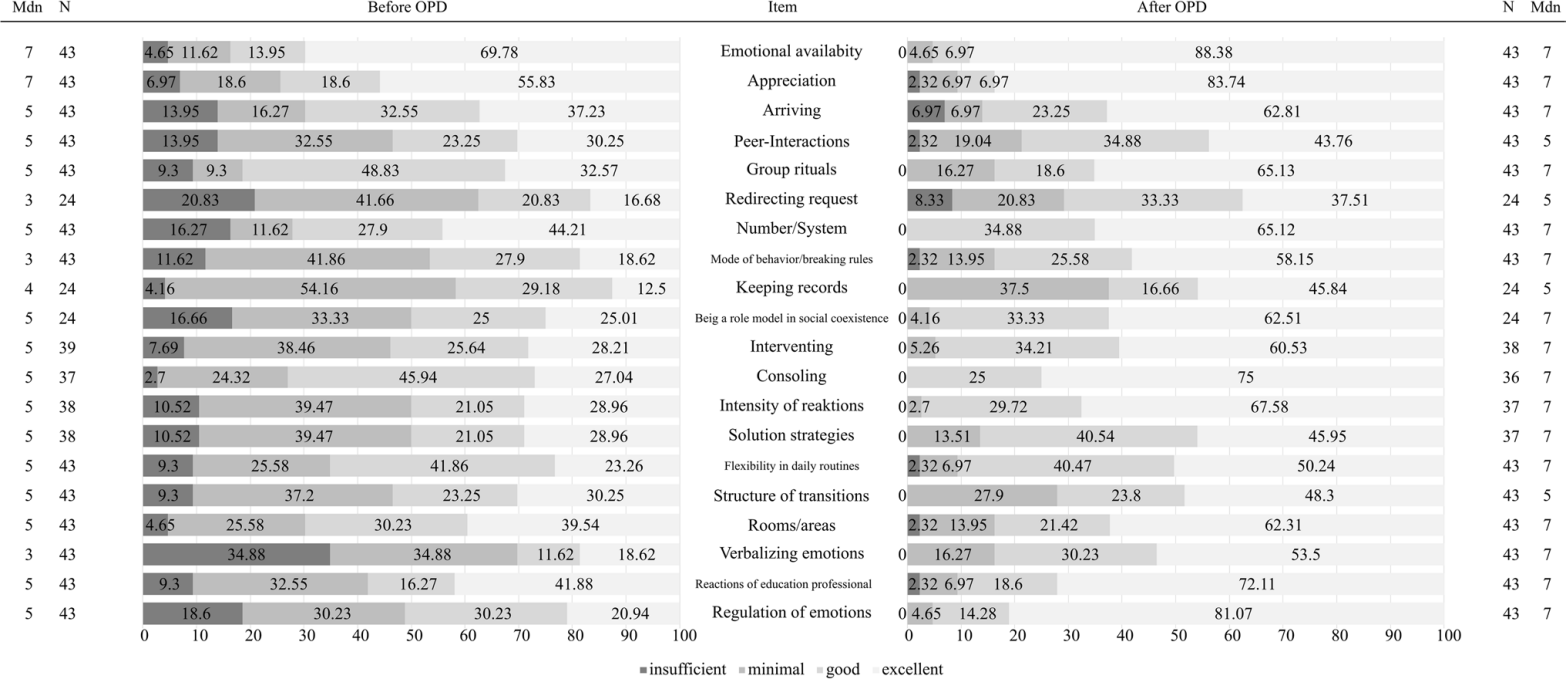
Differences in the central tendency before and after attending the online professional development programme: subscale 'Ensure relationships and well-being'. Subscale 'Ensure relationships and well-being' (before OPD: Mdn = 5; IQR = 4; Cronbachs Alpha = 0.892; after OPD: Mdn = 7; IQR = 2; Cronbachs Alpha = 0.828), number of participants

**Fig. 4:**
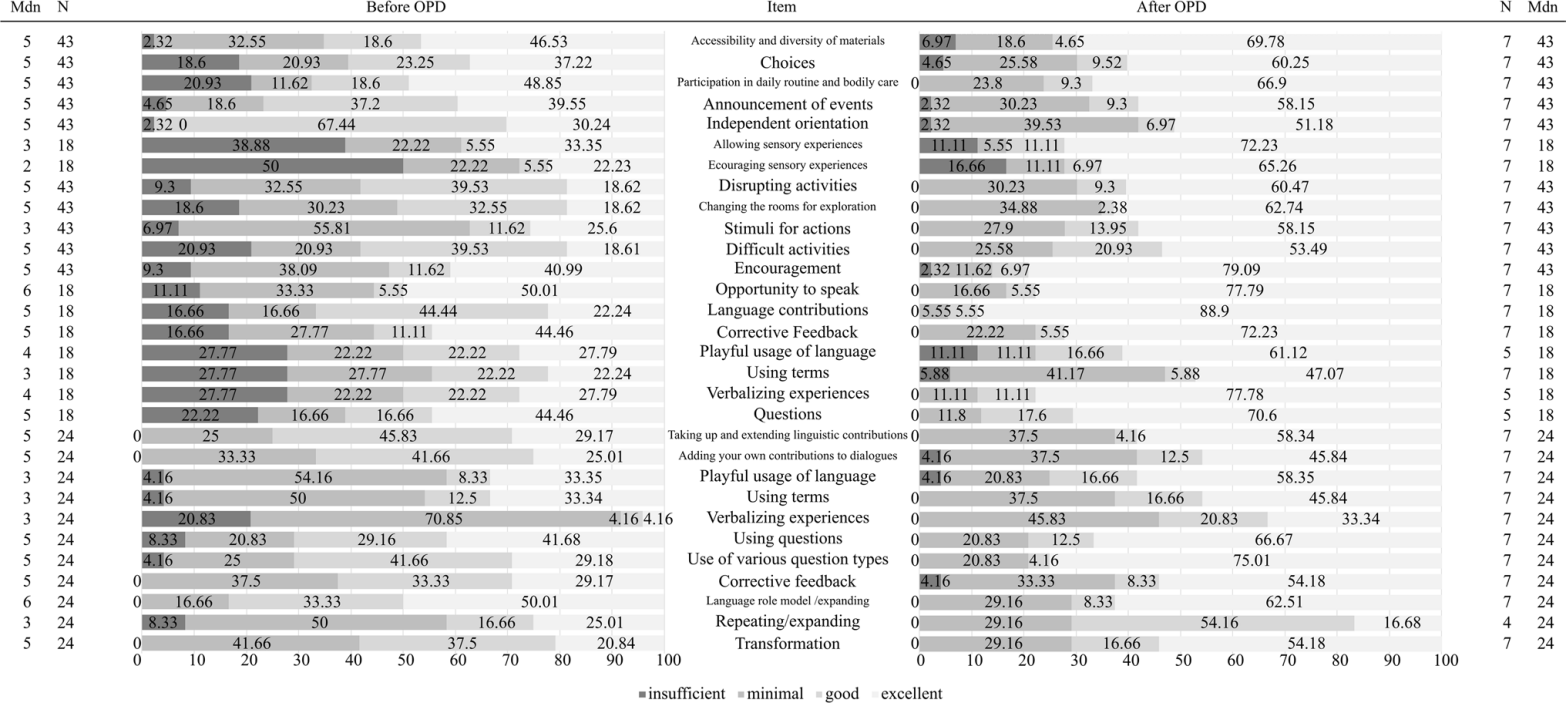
Differences in the central tendency before and after attending the online professional development programme: subscale 'Support learning'. Subscale 'Support learning' (before OPD: Mdn = 5; IQR = 2; Cronbachs Alpha = 0.966; after OPD: Mdn = 7; IQR = 2; Cronbachs Alpha = 0.892), number of participants

Figures 3 and 4 show the descriptive statistics of the assessments before and after completing the OPD programme. It should be noted that the significance of group differences between the individual aspects was not assessed because the risk of increasing the alpha error probability due to multiple testing was extremely high (Dormann, 2017).

As the median values in Figs. 3 and 4 show, quality improved after completion of the OPD programme for most (90%) of the criteria but remained unchanged for individual aspects (10%). A detailed analysis of the quality measurement data before and after completion of the OPD programme showed that 26 and 32 participants, respectively, improved the quality of their interactions with children in the subscales 'Ensure relationships and well-being' and 'Support learning'. In the former subscale, the quality of interactions with children remained the same for 15 participants (11 of whom had been evaluated as excellent before completing the OPD course), while the quality deteriorated by one level for two participants. In the latter subscale, the quality of interactions with children remained the same for 11 participants (six of whom had been evaluated as excellent before completing the OPD course).

The significance of the changes between the first and the second measurement was assessed using the Wilcoxon test (Table 4).

**Table 4:** Wilcoxon test before/after

Subscale	*Mdn* before OPD	*Mdn* after OPD	*P*	*Z*
'Ensure relationships and well-being'	5	7	0.000	− 4.524^a^
'Support learning'	5	7	0.000	− 4.898^a^

^a^Based on negative scores

The Wilcoxon test showed that the quality of interaction improved significantly in the subscale 'Ensure relationships and well-being' after attending the training course (*Mdn*_before_ = 5, *Mdn*_after_ = 7; asymptotic Wilcoxon test: *Z* =  − 4.524, *p* = 0.000; *r* = 0.68). In the subscale 'Support learning', there was a statistically significant difference in the quality of interaction between the two measurements (*Mdn*_before_ = 5, *Mdn*_after_ = 7; asymptotic Wilcoxon test: *Z* =  − 4.898, *p* = 0.000; *r* = 0.74).

## Discussion and Limitations

The results of the quality measurements before and after completion of the OPD programme show a significant improvement in performance with a focus on the quality of interactions between early childhood professionals and children. This is in line with other empirical studies showing that training programmes that address personal situations are effective (Egert & Kappauf, 2019; Egert et al., 2017). However, the study has some limitations: One of them is the long interval (almost one year) between the first and second quality measurement. During this period, besides attending the OPD training, the participants may have gained further professional experience or even received additional training beyond the OPD programme. The second limitation is the one-group-pretest–posttest design without a control group. Thus, there is no evidence that the OPD blended learning course had a direct impact on the observed quality of interactions in practice. However, the data were generated through standardised before/after observations and are not based on participants’ self-reports. The quantitative data can be interpreted as a first evaluation of an online professional development programme. The third limitation is, that the socio-demographic data of the participants was not collected uniformly. Thus, no conclusions can be drawn about other characteristics (e.g.: work experience, basic education, etc.). In the case of OPD courses that include theoretical modules, where there is a need for modules that interest participants personally and a need to put theory into practice, an individualised format can contribute to the success of OPD programmes. This is consistent with the findings of Scarinci et al. (2015). Other factors contributing to the success of the OPD course may be the different methods used in the theoretical modules and the reflections sessions in small groups (8 to 12 participants). For further studies, it would be interesting to collect more detailed socio-demographic data of the participants in order to be able to draw further conclusions.

## Conclusions and Recommendations

The advantage of this blended learning format is its flexible use. It is a practical means for participants to further their education, as they can freely allocate most of their time. Moreover, they can participate from different regions and countries. Therefore, there are no costs or travel times involved. In addition, making the content available in several languages can help overcome language barriers.

Empirically verifiable quality assurance and development through standardised measurements is further evidence that CPD formats—in this case, a blended learning CPD format—can increase the professionalisation of early childhood educators. Criteria for success can be the different methods, the individual, needs-based use of theoretical input and reflection sessions in small learning communities of 8 to 12 participants, which can facilitate a solid transfer from theory to practice. Another advantage is that the effectiveness of the various quality features is directly observable in the interactions between professionals and children. In this way, the children can benefit from proven quality assurance and development. Apart from the individual quality measurements on site, the blended learning format is cost-efficient for the participants as they save time and money (petrol, parking fees) for travelling. Since people in remote areas can easily participate in these formats, the OPD format could be used as a measure for professionalising ECE in times of a shortage of specialists, which can be multiplied and also be deployed quickly in terms of time. In view of the prevailing shortage of skilled professionals, it is interesting to think about new training and further education mechanisms, as well as certification and qualification processes oriented towards the proven—and observable—competencies of early childhood professionals.
